# Three-dimensional relationships between condylar volume and dentoskeletal characteristics in Class II hyperdivergent female adults

**DOI:** 10.1186/s12903-023-02838-x

**Published:** 2023-03-13

**Authors:** Bingjie Xie, Lingyi Huang, Anna Feng, Xianglong Han, Ye Tian, Juan Li

**Affiliations:** 1 State Key Laboratory of Oral Diseases, National Clinical Research Center for Oral Diseases, West China Hospital of Stomatology, Sichuan University, No 14, 3Rd Section, Renmin Nan Road, Chengdu, 610000 China; 2 University of Texas Health Science Center at Houston, Taxes Houston, USA

**Keywords:** Three-dimensional analysis, Class II hyperdivergent, Temporomandibular disorders, Condylar morphology, Dentofacial characteristics

## Abstract

**Background:**

This study aimed to determine the differences among various volumes of condylar osseous patterns and the corresponding dentoskeletal characteristics based on the risk of temporomandibular disorder.

**Methods:**

Craniofacial spiral computed tomography data of 60 Class II hyperdivergent female adults were divided into normal, resorptive, flattened, and osteophyte groups based on condylar osseous forms. The condylar volumes of each group were compared, and their correlations with the dentoskeletal characteristics were assessed in three dimensions. Pairwise least significant difference tests were used to examine individual pairwise differences between groups, and one-way analysis of variance was used to measure differences among multiple groups. Pearson correlation and Spearman rank correlation analyses were used to determine the correlation between condylar volume and dentofacial characteristics. Statistical significance was established at *p* < 0.05.

**Results:**

The condylar volume in the normal group was significantly greater than that in the changed groups, with no significant differences between the subgroups. The decrease in condylar volume was associated with a retruded and clockwise-rotated mandible with shorter rami. Condylar volume was negatively correlated with overjet, the alveolar height of the lower anterior and posterior teeth, sagittal inclinations of the lower teeth, intermolar width of the mandibular first molars, and width between the corresponding alveolar crests.

**Conclusion:**

Multiple three-dimensional dentoskeletal characteristics of Class II hyperdivergent female adults are correlated with condylar bony changes, regardless of the form. These results could be helpful in indicating potential pathological changes in the temporomandibular joint and in making proper treatment plans for these patients.

## Background

The morphology of the temporomandibular joint (TMJ) is associated with the dynamic balance of mandibular function and can affect the relationship between the maxilla and mandible in all dimensions. As an important part of the TMJ, the condyle is of great importance for the long-term stability of orthodontic and orthognathic treatment and should be considered in treatment planning [1–3].

Condylar morphology may be determined by the different loading modes of various craniofacial morphologies [4]. In addition to physiological factors, several pathological conditions, such as temporomandibular disorders (TMDs) [5], idiopathic condylar resorption [6], and rheumatoid arthritis [7], could influence condylar volume and shape. These remodeling or degenerative changes in the condyle include erosion, resorption, flattening, sclerosis, and osteophyte formation [8]. Therefore, measurements of condylar morphology may be helpful in identifying risk factors for some pathologies.

Although there is some controversy [9, 10], condyle morphology is thought to be associated with craniofacial and occlusal features [11–14]. Moreover, degenerative changes in the condyle may be linked to some dentofacial characteristics [15]. However, no studies have compared the differences among various bony change forms of the condyle, and the correlation between dentoskeletal characteristics and the forms of condylar osseous alteration has not been explored.

Traditional studies have used lateral cephalograms to study sagittal and vertical dentofacial structures [8, 15, 16], which increased the possibility of error caused by superimposition of neighboring structures. Additionally, little is known about the association between condylar features and transverse skeletal and occlusal conditions. Although some studies have attempted to assess condylar morphology using computed tomography (CT), most measurements have been conducted in two dimensions (2D) [17–20]. Hence, it is imperative to use a 3D approach to more accurately evaluate the relationship between condylar osseous changes and dentofacial modalities.

The aims of this retrospective study were to compare the condylar volume among condyles with different morphological bony changes and determine the relationship between condylar volume and dentofacial characteristics in 3D using CT. To minimize bias, the participants in this study were confined to skeletal Class II hyperdivergent female adults.

## Methods

The sample consisted of 60 subjects who had undergone craniofacial spiral CT scans at the X hospital for any reason from June 2018 to April 2021. Samples were selected based on the following inclusion criteria: (1) females aged 20–40 years; (2) skeletal Class II, ANB angle > 5°; (3) hyperdivergent profile (SN-MP angle > 40°, FMA angle > 32°, posterior/total facial height ratio > 65%); (4) no facial asymmetry (menton deviates from midsagittal plane less than 2 mm); (5) no history of cleft lip or palate, craniofacial syndrome, trauma, orthodontic treatment, or surgery; (6) fully erupted permanent teeth and no malformed, ectopic erupted, or missing teeth; (7) no crowns, implants, brackets, or cuspal restorations; (8) no apparent crowding (< 5 mm per arch); (9) no cross bite; (10) if condylar bony changes exist, the changes should be seen in at least 2 consecutive slides of CT images and the cortical bone of the condylar surface should be continuous [20]. Patients with Class II division 2 were excluded.

**Fig. 1:**
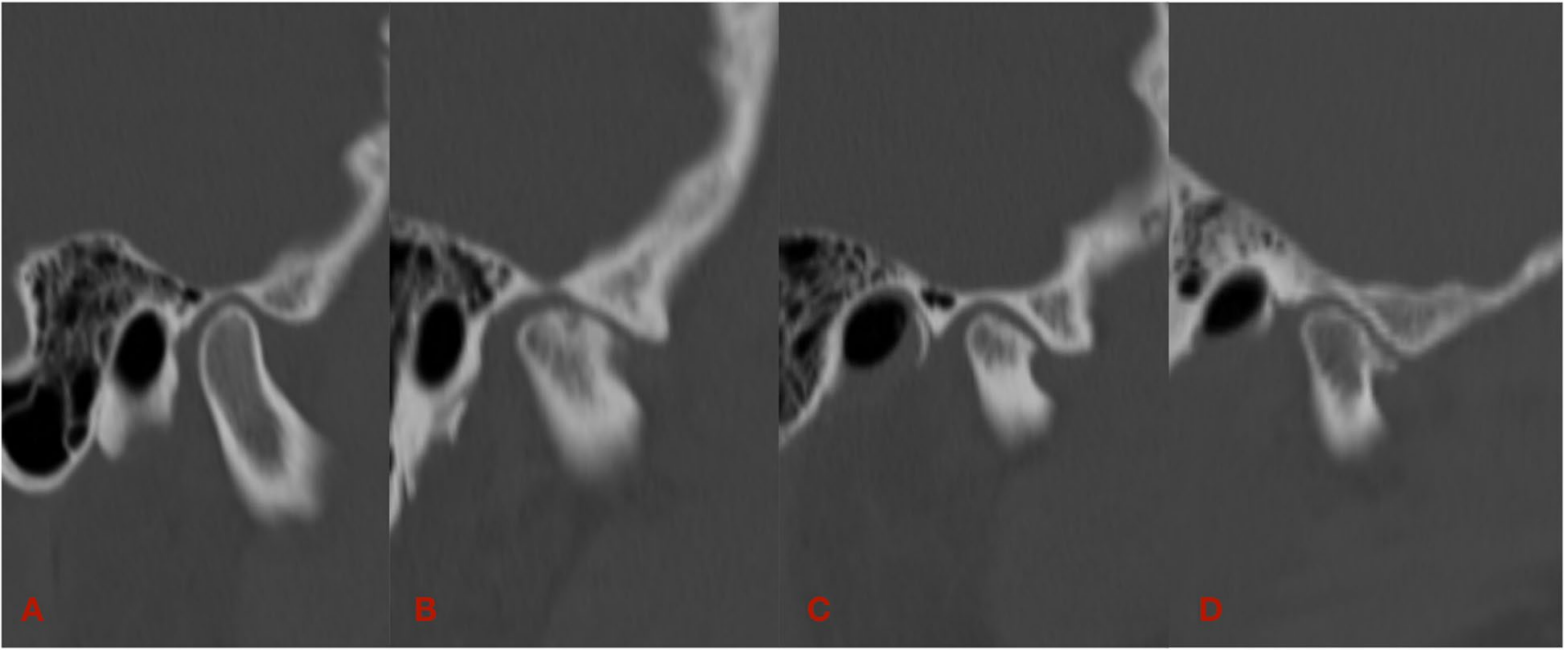
Sagittal view of four different types of condylar head bony morphologies: **A**, normal; **B**, resorption; **C**, flattening; **D**, osteophyte

The subjects were divided into normal and changed groups. The normal group included 15 subjects with no obvious abnormal bony morphological changes on either side of the condyle. The samples of the changed group were defined as condyles with evident bilateral identical bony changes. The changed group was further subdivided into resorption, flattening, and osteophyte groups (Fig. 1). The resorption group had 15 subjects, including partial bone loss other than surface flattening on the condylar heads. The flattening group had 16 subjects with a flat bony contour that deviated from the normal convex forms of the condyles. The osteophyte group had 14 subjects manifesting as a marginal bony outgrowth on the condyles [8].

**Fig. 2:**
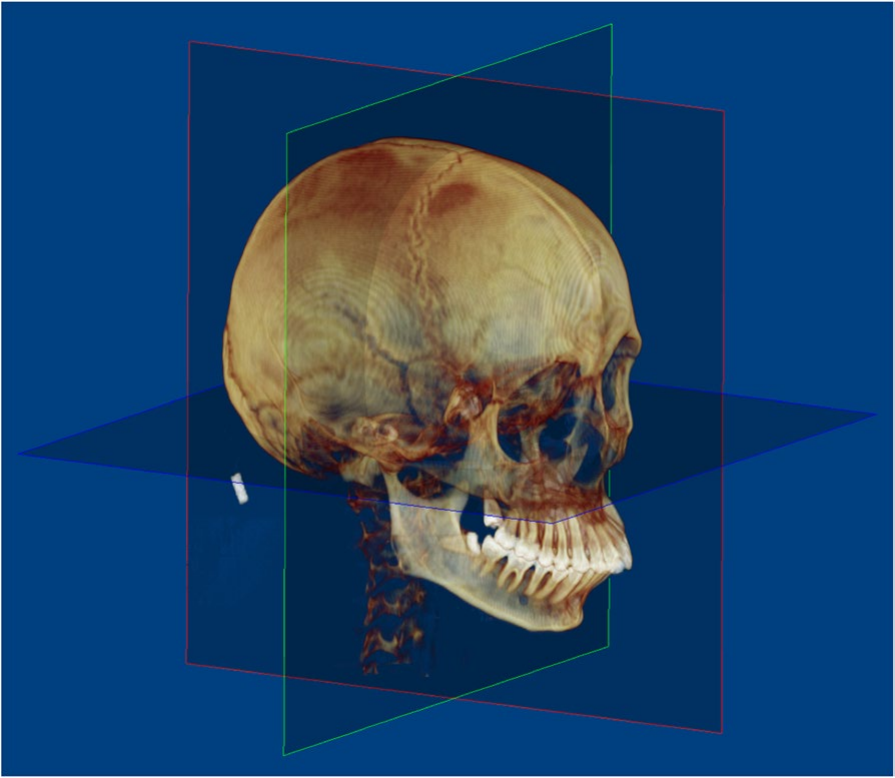
Reorientation based on reference planes. Blue: axial plane, FH plane; red: midsagittal plane, perpendicular to axial plane, passing through nasion and basion; green: coronal plane, perpendicular to the axial and coronal planes, passing through bilateral porions

The CT equipment used was a Philips MX 16-slice system (Philips Healthcare, Best, Netherlands), and the images were obtained at 90 kV, 40 mA, and a voxel size of 0.49 mm. Images were saved as Digital Imaging and Communication in Medicine (DICOM) files and reconstructed in Dolphin software (version 11.7; Dolphin Imaging and Management Solutions, Chatsworth, CA). Reorientation of each scan was performed using the standardized head position (Fig. 2) [20].

**Fig. 3:**
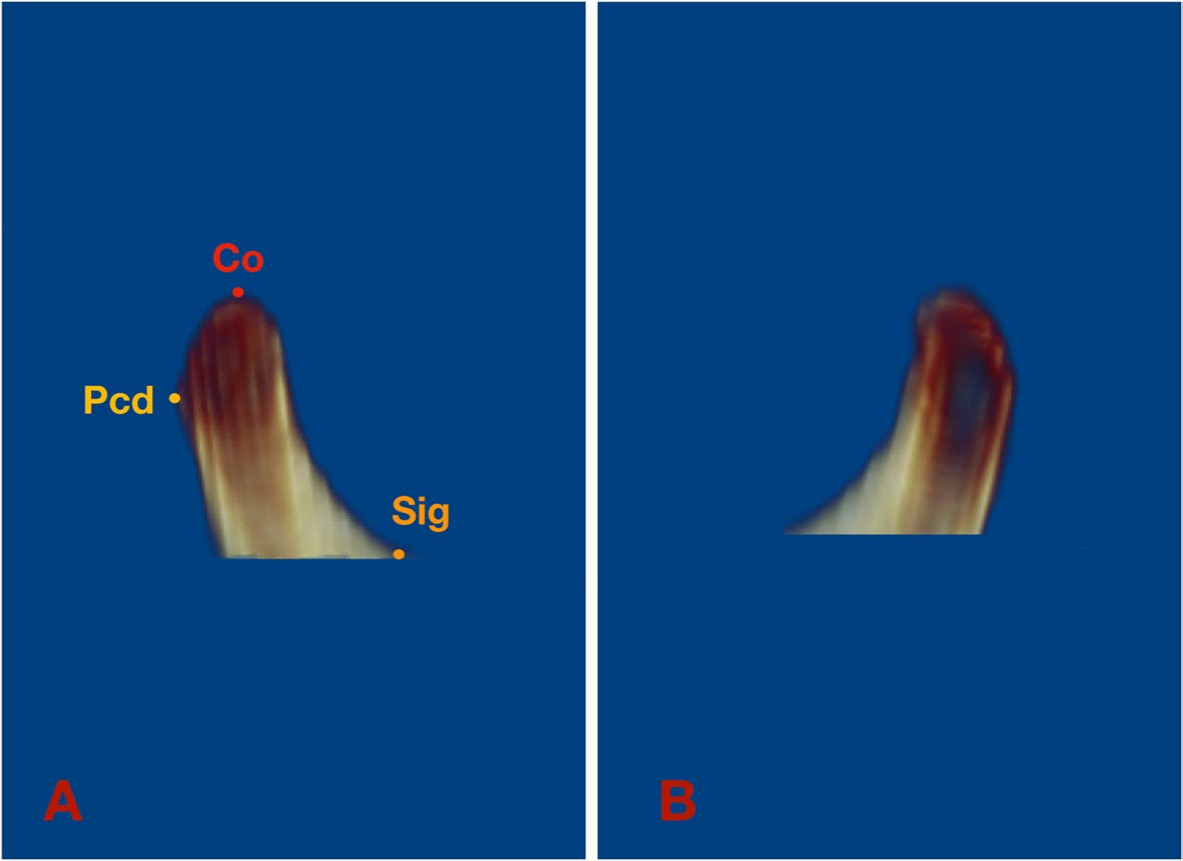
Sagittal view of unilateral condyle. **A**: right condyle; **B**: left condyle. Condylion (Co): most superior point of condyle; Pcd: Posterior condyle point; Sig: most inferior point of sigmoid notch

The Hounsfield value (HU) set was assigned to 176–2476 by a built-in tool of Dolphin in order to standardize 3D rendering of the condyles for further volumetric evaluation. The boundary of the surface outline of condyles could be distinguished by the color map of the HU set. The unilateral condyle was separated from the skull by clipping and sculpting in Dolphin. The following planes were used to define the borders of the condyles: a plane tangent to the condylion (Co) point and parallel to the axial plane was used as the superior boundary, a plane tangent to the most inferior point of the sigmoid notch and parallel to the axial plane served as the inferior boundary, a plane passing through the deepest point of the sigmoid notch and perpendicular to the axial plane was determined as the anterior border, and a plane tangent to the most posterior point of the condyle (Pcd) and perpendicular to the axial plane was characterized as the posterior border (Fig. 3) [21]. Once the condyle was isolated, the condylar volume in each group was measured using Dolphin software.

**Fig. 4:**
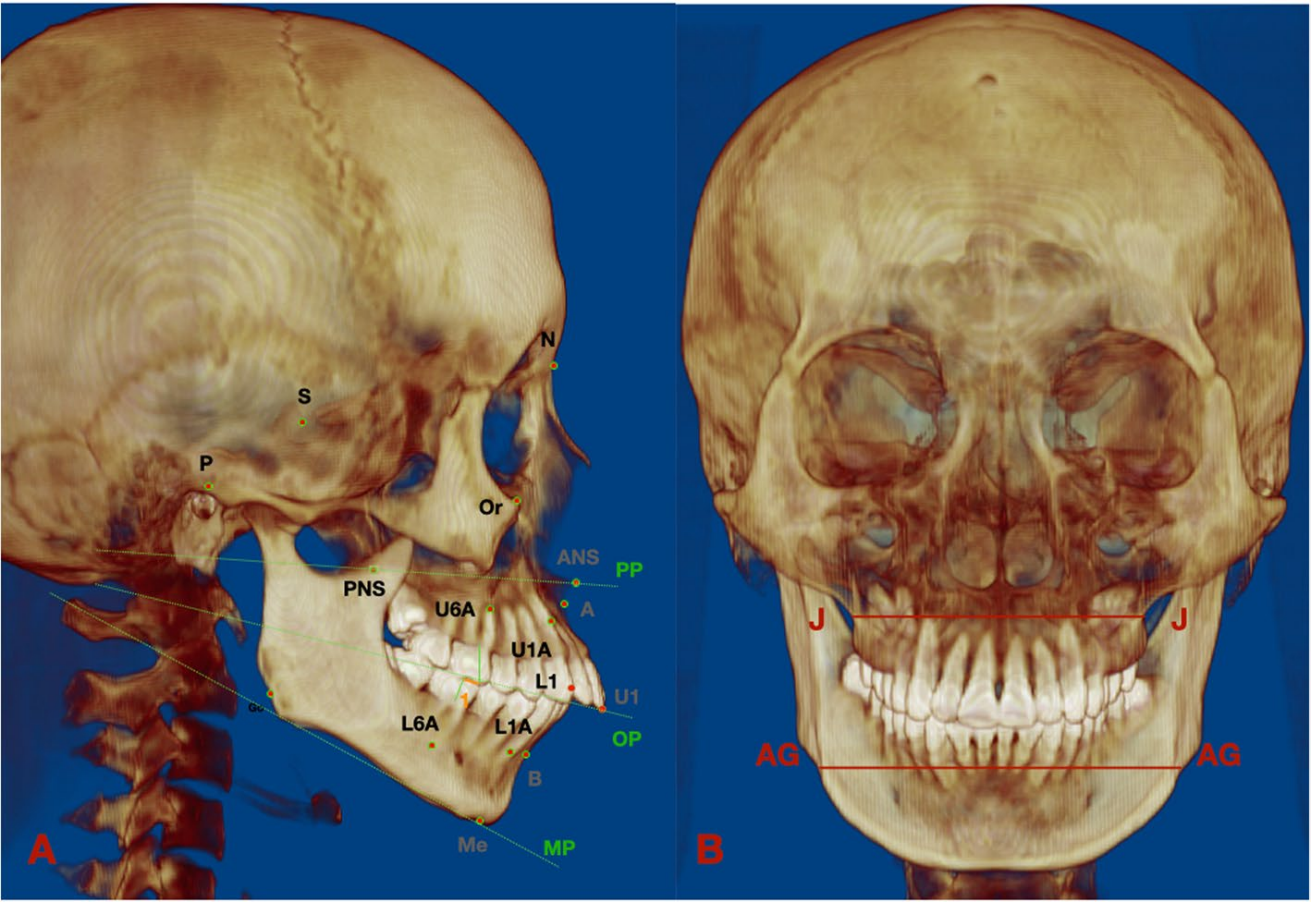
Landmarks, planes and measurements on the 3D reconstruction. **A**: the sagittal view: 1: 6-MD. **B**: the frontal view

**Fig. 5:**
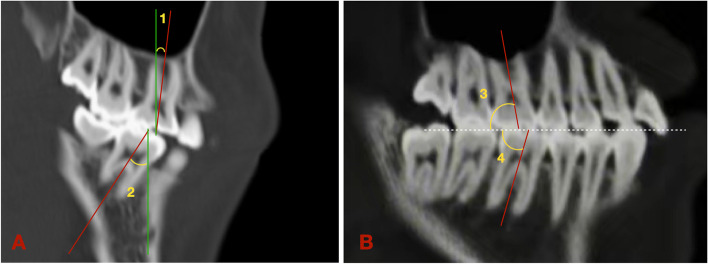
Angular measurements of right first molars on the sagittal CT image. Red line: the long axes of upper/lower first molar. Green line: planes parallel to the coronal plane. White dot line: occlusal plane. **A**: the angle between first molars and the coronal plane. 1: R: ∠16/Coronal; 2: R: 46/Corona. **B**: the angle between first molars and the occlusal plane. 3: Sagittal: ∠16/OP; 4: Sagittal: ∠46/OP

**Fig. 6:**
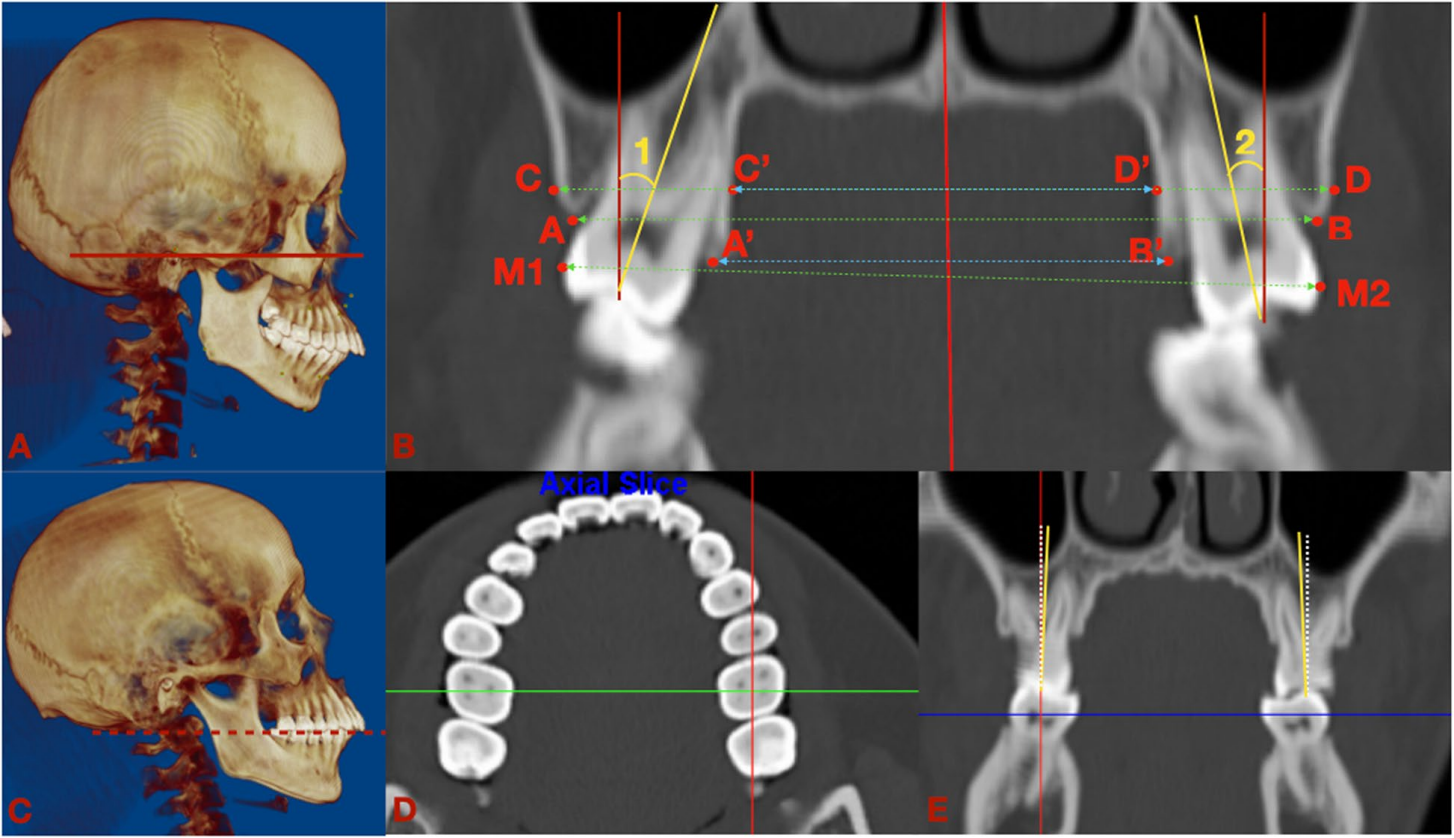
The demography of transverse measurements on the upper first molar. **A**: measured when the FH plane was parallel to the floor; **B**: Linear and angular measurements of upper first molar on the coronal CT image. **A**-**B**: U6-AC; **C**-**D**: U6-MR; M1-M2: U6-IM; (AB-A’B’)/2: U6-BLW; (CD-C’D’)/2: U6MR-BLW; 1: ∠16/Sagittal; 2: L: ∠46/Sagittal; **C**: measured when the OP was parallel to the floor; **D**:on the axial section of maxillary arch, the coronal slide was defined as a line (green line) connecting the midpoint of the mesiodistal occlusal crown width of bilateral first molars; **E**: the long axis of upper first molar was defined as a line (yellow line) passing through the midpoint of the buccal and lingual cusp tips and the midpoint of the buccolingual width at the cervical base close to the furcation of the anatomic crown. The BLI of the tooth was the angle formed by the long axis of molar and true vertical line

**Fig. 7:**
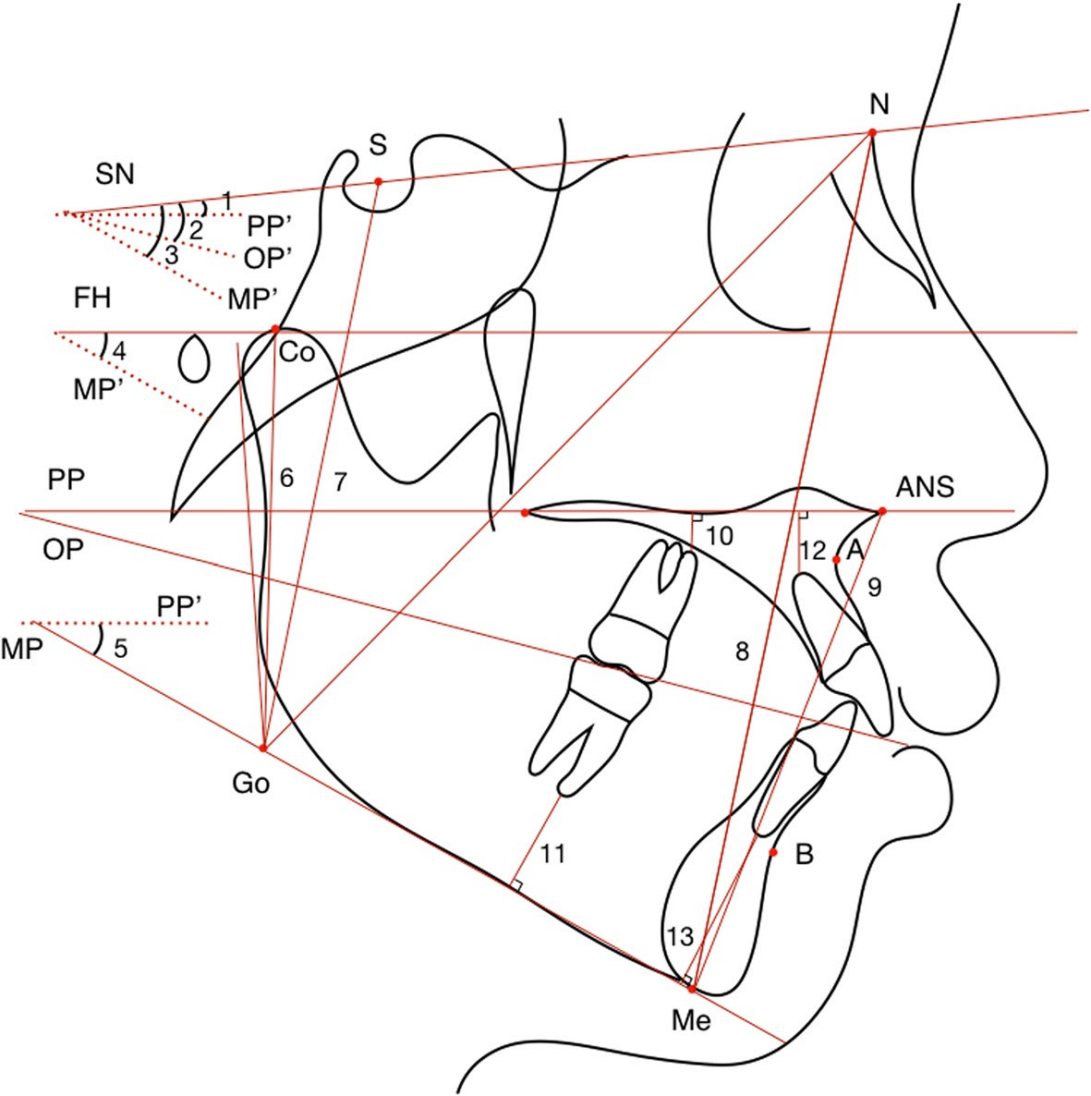
Parts of vertical variables. 1: ∠SN-PP; 2: ∠SN-OP; 3: ∠SN-MP; 4: ∠FMA; 5: ∠PP-MP; 6: Co-Go; 7: S-Go; 8: N-Me; 9: ANS-Me; 10: U6-PP; 11: L6-MP; 12: U1-PP; 13: L1-MP

**Fig. 8:**
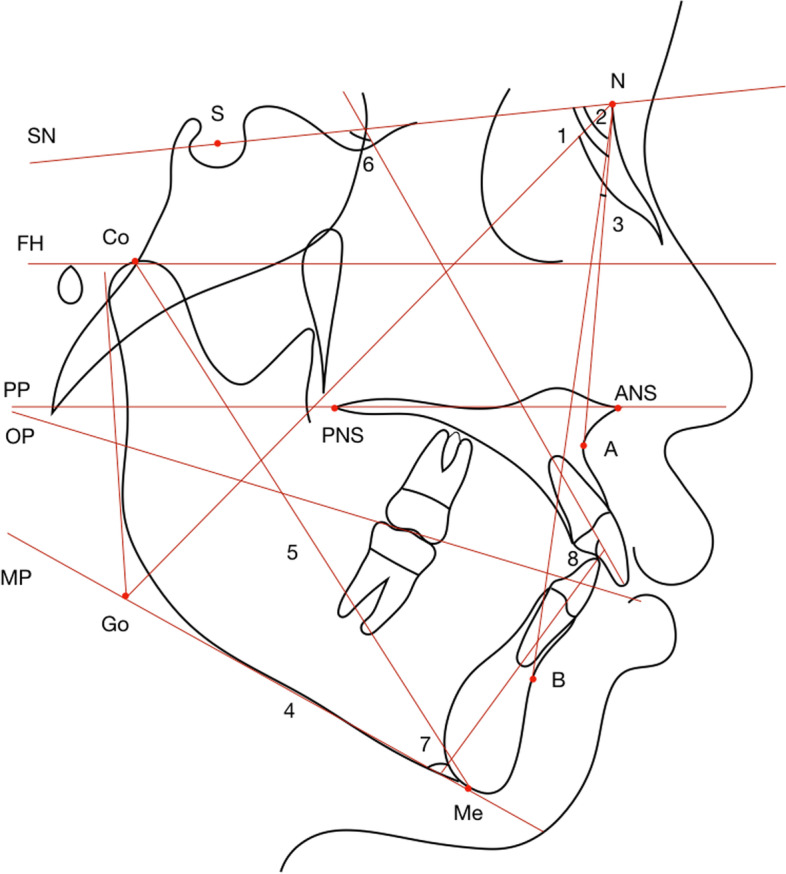
Parts of sagittal variables. 1: ∠SNA; 2: ∠SNB; 3: ∠ANB; 4: Go-Me; 5: Co-Me; 6:∠U1-SN; 7:∠IMPA; 8:∠U1-LI

Table 1 lists the landmarks, plans, and measurements utilized for the analyses. Skeletal and dentoalveolar variables were measured on CT or converted images (Figs. 4, 5, 6, 7 and 8). Lateral cephalograms were made from CT imaging based on the left and right halves of the reconstructed skull. Cephalometric variables were measured separately for each half, and averages were calculated and recorded.

**Table 1:** Definition of points, measurement planes and measurements

**Landmarks**	**Definition**
N	Nasion
S	Sella
Or	Orbitale
P	Porion
ANS	Anterior nasal spine
PNS	Posterior nasal spine
A	Point A
U1	Incisal edge of maxillary incisor
U1A	Apex of maxillary incisor
L6A	Apex of mandibular first molar
B	Point B
Me	Menton
Go	Gonion
J	Interjugular point
AG	Antegonial notch point
**Measurement planes**	**Definition**
FH. Frankfort horizontal plane (Fig. 2)	Plane passing through bilateral porions and the midpoint of bilateral orbitales
Midsagittal plane (Fig. 2)	Plane perpendicular to FH plane, passing through nasion and basion
SN	Sella nasal plane
PP. Palatal plane	Perpendicular to the sagittal plane, passing through ANS and PNS
OP. Occlusal plane	Functional occlusal plane, along the maximum intercuspation of the posterior teeth
MP. Mandibular plane	Passing through Me, tangent to the lower border of mandible
**Measurements**	**Definition**
L Volume	The volume of the left condyle
R Volume	The volume of the right condyle
**Measurements of** Fig. 4	**Definition**
6-MD	Sagittally discrepancy of both arches, the distance between the projection of mesiobuccal cusp of upper first molar and mesiobuccal groove of lower first molar to the OP
J-J	Maxillary width, transverse width at the bilateral interjugular point
AG–AG	Mandibular width, transverse width at the antegonial notch point
MxMn difference	The difference between the AG–AG and J-J width
**Measurements of** Fig. 5	**Definition**
∠16/Coronal	The angulation of the angle formed by the long axes of right upper first molar and the coronal plane
Sagittal: ∠16/OP (°)	The angulation of the angle formed by the long axes of right upper first molar and the occlusal plane on the sagittal view
**Measurements of** Fig. 6	**Definition**
U6-AC	Transverse width at the alveolar crest of bilateral maxillary first molar
U6-MR	Transverse width at the alveolar crest of the midpoint of bilateral maxillary first molar root length
U6-BLW	Buccolingual width at the alveolar crest of bilateral maxillary upper first molar, the difference between buccal and lingual AC transverse widths divided by 2
U6MR-BLW	Buccolingual width at the alveolar crest of the midpoint of bilateral maxillary upper first molar root length, the difference between buccal and lingual AC transverse widths at midpoint root length divided by 2
U6-IM	Transverse width between the most prominent points on the buccal surface of bilateral maxillary first molar
∠26/Sagittal	The angulation of the angle formed by the long axes of left upper first molar and the sagittal plane
Coronal: ∠16/OP (°)	The buccolingual inclination of right upper first molar
**Measurements of** Fig. 7	**Definition**
U6-PP	Distance between the mesiobuccal root apex of upper first molar and PP
L6-MP	Distance between the mesiobuccal root apex of lower first molar and MP
U1-PP	Distance between the incisal edge of upper central incisor and PP
L1-MP	Distance between the incisal edge of lower central incisor and MP
Co-Go	Ramus height
S-Go	Total posterior facial height
N-Me	Total anterior facial height
ANS-Me	Lower anterior facial height
U6-PP	Distance between the mesiobuccal root apex of upper first molar and PP
L6-MP	Distance between the mesiobuccal root apex of lower first molar and MP
U1-PP	Distance between the root apex of upper central incisor and PP
L1-MP	Distance between the root apex of lower central incisor and MP
**Measurements of** Fig. 8	**Definition**
Go-Me	Mandibular body length
Co-Me	Total mandibular length
∠U1-SN	Maxillary incisor to SN plane angle
∠IMPA	Mandibular incisor to mandibular plane angle
∠U1-LI	Interincisal angle

### Statistical analysis

All statistical analyses were performed using SPSS software (version 20.0; IBM, Armonk, NY). Two orthodontists with more than 5 years of experience independently evaluated the forms of condyles and labeled involved landmarks. A senior orthodontic professor was consulted in case of disagreement. After a 2-week interval, 20 subjects were randomly selected and remeasured for all variables. The intraclass correlation coefficient (ICC) was calculated to examine intra-observer reliability, while the first and last measurements were used to examine interobserver reliability. For continuous variables, the Shapiro -Wilk test was performed to test whether the variables of each group followed a normal distribution. If the variables followed a normal distribution, independent t-tests were performed to detect the difference within each group, while Mann–Whitney U test was used if the data was abnormally distributed. Analysis of variance (ANOVA) was employed to compare continuous variables among the different groups. If the continuous variables did not follow a normal distribution, the Kruskal–Wallis test was used and was further corrected by the Bonferroni test. Post-hoc analysis was performed when statistical significance was detected. Pearson correlation and Spearman rank correlation analyses were used to determine the correlation between condylar volume and dentofacial characteristics. Linear regression analysis was performed to establish the regression equation. Statistical significance was set at *p* < 0.05.

## Results

The ICC values ranged from 0.916 to 0.921 for intra-observer reliability and 0.907 to 0.931 for interobserver reliability, indicating satisfactory reproducibility of these measurements. There was no significant difference between the condyles on the left and right sides within each group (*p* > 0.05) (Table 2). Therefore, the average volumes of the left and right condyles were used for further analysis.

**Table 2:** Comparison of bilateral condylar volume differences among each group

Group	L Volume(mm^3^)	R Volume(mm^3^)	T/Z	*p*
All	1301.11 ± 465.11	1334.74 ± 447.82	-0.514	0.607^#^
Normal	1798.08 ± 295.04	1768.73 ± 227.69	0.305	0.763
Resorption	1244.73 ± 383.05	1319.48 ± 421.28	-0.508	0.615
Flattening	1056.23 ± 284.53	1098.58 ± 262.01	-0.438	0.665
Osteophyte	1108.93 ± 484.14	1155.99 ± 513.18	-0.551	0.581^#^

Independent t test were used since the data was normally distributed

^#^: Mann–Whitney U test was used in this group since the data was abnormally distributed

Values do not differ significantly (*p* > 0.05)

The condylar volume was significantly higher in the normal group than in the other three changed groups (*p* < 0.05). However, pairwise comparisons showed no significant differences among the changed groups (*p* > 0.05) (Table 3). Hence, we decided to focus on analyzing the relationship between condylar volume and dentofacial characteristics.

**Table 3:** Comparison of the condylar volume differences between four groups

Group	L Volume(mm^3^)	R Volume(mm^3^)	Average Volume(mm^3^)
Normal (I)	1816.79 (1465.84,2018.87)	1796.68(1565.32,1996.2)	1824.25(1565.9,1956.54)
Resorption (II)	1195.67 (871.66,1453.97)*	1368.34(954.03,1568.72)*	1150.07(926.32,1465.14)*
Flattening (III)	1087.05 (910.24,1236.94)***	1136.66(977.94,1290.04)***	1150.39(936.83,1222.53)***
Osteophyte (IV)	964.38 (785.02,1274.31)**	1066.33(838.28,1223.41)**	988.6(835.61,1248.86)***
C^2^	21.159	20.242	21.741
*p*	< 0.001	< 0.001	< 0.001

Results of post-hoc analysis

L Volume: I vs II *p<0.05, I vs III ***p<0.001, I vs IV **p<0.01, II vs III *p*＞0.1, II vs IV *p*＞0.1, III vs IV *p*＞0.1

R Volume: I vs II *p<0.05, I vs III ***p<0.001, I vs IV **p<0.01, II vs III *p*＞0.1, II vs IV *p*＞0.1, III vs IV *p*＞0.1

Average Volume: I vs II *p<0.05, I vs III ***p<0.001, I vs IV ***p<0.001, II vs III *p*＞0.1, II vs IV *p*＞0.1, III vs IV *p*＞0.1

In the vertical plane, the condylar volume was positively correlated with Co-Go, S-Go, S-Go/N-Me and negatively correlated with the SN-OP, SN-MP, FMA, PP-MP, U6-PP, L6-MP, and L1-MP angles (*p* < 0.05) (Table 4).

**Table 4:** Comparison of vertical measurements and measurements related to condylar volume

Variables	Normal	Pathological			r	*p*
		**Resorption**	**Flattening**	**Osteophyte**		
∠SN-PP (°)	9.51 ± 1.02	10.33 ± 0.85	10.41 ± 0.71	11.09 ± 0.81	-0.043	0.745
∠SN-OP (°)	21.84 ± 0.98	25.42 ± 1.08	27.58 ± 1.50	25.06 ± 1.22	-0.394	0.002*
∠SN-MP (°)	42.39 ± 0.92	47.82 ± 0.75	48.97 ± 2.07	48.56 ± 1.48	-0.423	0.001*
∠FMA (°)	30.44 ± 0.81	34.84 ± 1.02	35.71 ± 1.71	35.03 ± 1.29	-0.509	0.000*
∠PP-MP (°)	33.25 ± 1.36	33.99 ± 1.16	38.41 ± 2.06	37.87 ± 1.52	-0.436	0.001*
Co-Go (mm)	59.63 ± 1.13	56.21 ± 1.45	51.92 ± 1.06	51.87 ± 1.70	0.765	0.000*
S-Go (mm)	80.77 ± 1.51	78.39 ± 1.25	75.1 ± 1.76	72.91 ± 1.72	0.573	0.000*
N-Me (mm)	129.19 ± 1.49	128.29 ± 1.01	130.21 ± 1.43	129.68 ± 1.75	0.249	0.057
ANS-Me (mm)	75.56 ± 1.09	74.19 ± 0.93	76.57 ± 1.35	76.49 ± 1.58	0.181	0.171
S-Go/ N-Me	0.63 ± 0.01	0.61 ± 0.01	0.58 ± 0.01	0.56 ± 0.01	0.466	0.000*
ANS-Me/ N-Me	0.58 ± 0.01	0.58 ± 0.01	0.59 ± 0.01	0.59 ± 0.01	-0.024	0.855
U6-PP (mm)	6.83 ± 0.38	6.16 ± 0.52	5.68 ± 0.36	5.95 ± 0.6	-0.449	0.000*
L6-MP (mm)	16.06 ± 0.57	15.67 ± 0.6	16.16 ± 0.91	15.34 ± 0.84	-0.337	0.009*
U1-PP (mm)	11.03 ± 0.71	10.51 ± 0.5	10.4 ± 0.46	10.09 ± 0.44	0.035	0.792
L1-MP (mm)	25.37 ± 0.83	23.51 ± 0.63	24.71 ± 1.03	23.25 ± 1.04	-0.308	0.018*
Overbite (mm)	4.46 ± 0.5	2.65 ± 0.71	2.06 ± 0.81	2.73 ± 0.74	0.203	0.124

*p < 0.05

In the sagittal plane, the condylar volume was positively correlated with the ∠SNB, Co-Me, and ∠U1-L1 and negatively correlated with ∠ANB, the sagittal inclination of the lower posterior teeth, ∠IMPA angle, and overjet (*p* < 0.05). There was no significant correlation with ∠SNA, Go-Me, or ∠U1-SN (Table 5).

**Table 5:** Comparison of sagittal measurements and measurements related to condylar volume

Variables	Normal	Pathological			r	*p*
		**Resorption**	**Flattening**	**Osteophyte**		
∠SNA (°)	81.26 ± 1.34	81.06 ± 1.27	81.53 ± 1.54	83.02 ± 1.18	0.426	0.285
∠SNB (°)	74.92 ± 1.75	74.81 ± 1.82	73.26 ± 1.37	71.73 ± 1.52	0.649	0.001*
∠ANB (°)	6.93 ± 0.48	6.97 ± 0.66	7.56 ± 0.43	9.02 ± 0.65	-0.328	0.011*
Go-Me (mm)	67.61 ± 1.12	67.63 ± 1.01	67.79 ± 1.43	67.92 ± 0.79	0.137	0.300
Co-Me (mm)	111.71 ± 1.21	109.03 ± 1.39	105.56 ± 1.01	98.86 ± 7.7	0.745	0.000*
L: 6-MD (mm)	1.93 ± 0.22	2.47 ± 0.35	1.81 ± 0.22	2.31 ± 0.33	-0.143	0.281
R: 6-MD (mm)	2.14 ± 0.2	2.9 ± 0.46	1.99 ± 0.28	2.94 ± 0.51	-0.137	0.302
∠27/Coronal (°)	-10.14 ± 2.71	-9.15 ± 2.73	-14.14 ± 3	-12.35 ± 2.38	0.501	0.257
∠26/Coronal (°)	-5.01 ± 1.92	-2.59 ± 1.72	-8.21 ± 2.38	-8.41 ± 1.23	0.363	0.395
∠37/Coronal (°)	30.64 ± 1.57	29.77 ± 2.17	34.68 ± 2.35	34.85 ± 1.81	-0.405	0.001*
∠36/Coronal (°)	27.84 ± 1.51	27.88 ± 1.9	30.76 ± 2.25	30.66 ± 1.26	-0.512	0.000*
∠17/Coronal (°)	-10.29 ± 2.4	-11.21 ± 3.2	-18.07 ± 2.77	-14.29 ± 2.05	0.436	0.171
∠16/Coronal (°)	-4.34 ± 2.24	-2.91 ± 1.43	-8.24 ± 2.77	-7.07 ± 1.17	0.441	0.380
∠47/Coronal (°)	28.99 ± 1.7	30.72 ± 2.5	32.79 ± 2.42	33.17 ± 1.64	-0.400	0.002*
∠46/Coronal (°)	25.85 ± 1.14	27.57 ± 2.16	32.16 ± 2.27	29.56 ± 1.38	-0.530	0.000*
Sagittal: ∠16/OP (°)	85.37 ± 2.81	86.81 ± 2.39	86.92 ± 2.62	86.79 ± 2.84	-0.137	0.296
Sagittal: ∠26/OP (°)	86.08 ± 2.42	86.39 ± 2.49	85.71 ± 3.03	87.74 ± 3.06	-0.018	0.894
Sagittal: ∠36/OP (°)	82.67 ± 3.67	73.99 ± 6.32	75.31 ± 3.65	76.98 ± 2.99	0.561	< 0.0001***
Sagittal: ∠46/OP (°)	83.04 ± 2.03	75.75 ± 3.27	76.57 ± 3.58	78.01 ± 2.45	0.516	< 0.0001***
∠U1-SN (°)	104.36 ± 2.18	106.25 ± 2.13	105.01 ± 2.18	105.36 ± 1.11	0.004	0.976
∠IMPA (°)	98.42 ± 1.6	107.12 ± 1.2	105.87 ± 1.11	106.16 ± 1.49	-0.321	0.035*
∠U1-L1 (°)	115.93 ± 2.84	108.07 ± 2.15	108.33 ± 1.34	108.15 ± 1.43	0.274	0.036*
Overjet (mm)	2.58 ± 0.37	4.32 ± 0.7	4.37 ± 0.53	4.47 ± 0.44	-0.376	0.003*

^*^p < 0.05

^***^p < 0.0001

In the transverse plane, the condylar volume showed a weak positive correlation with the buccolingual alveolar crest width of the maxillary first molar (U6-BLW) and a negative correlation with the angle between the mandibular first molar and the sagittal plane (∠L6/Sagittal) on both sides, the mandibular first molar arch width (L6-IM), and the mandibular transverse width at the alveolar crests of the first molars (L6-AC) (*p* < 0.05) (Table 6).

**Table 6:** Comparison of transversal measurements and measurements related to condylar volume

Variables	Normal	Pathological			r	*p*
		**Resorption**	**Flattening**	**Osteophyte**		
Max: J-J (mm)	64.19 ± 0.82	64.12 ± 0.88	64.76 ± 0.85	63.59 ± 0.52	-0.058	0.661
Man: AG–AG (mm)	84.1 ± 1.00	84.79 ± 1.75	86.96 ± 1.11	83.9 ± 53.68	-0.156	0.238
Man-Max (mm)	10.17 ± 0.53	10.36 ± 0.69	11.69 ± 0.34	10.06 ± 0.28	-0.061	0.647
U7-AC (mm)	61.12 ± 0.81	61.23 ± 0.8	61.96 ± 0.85	60.7 ± 0.77	-0.037	0.782
U7-MR (mm)	59.35 ± 4.12	63.2 ± 0.76	64.15 ± 0.95	62.64 ± 0.63	-0.017	0.900
U7-BLW (mm)	12.43 ± 0.82	11.29 ± 0.45	11.75 ± 1.16	12.36 ± 0.73	-0.117	0.378
U7MR-BLW (mm)	13.93 ± 0.47	10.85 ± 0.71	10.95 ± 0.84	10.45 ± 0.62	-0.023	0.861
U6-AC (mm)	57.44 ± 0.59	56.39 ± 0.83	54.54 ± 3.37	57.27 ± 0.61	-0.073	0.584
U6-MR (mm)	62.59 ± 0.79	61.33 ± 0.93	62.44 ± 0.81	61.24 ± 0.57	0.032	0.811
U6-BLW (mm)	11.54 ± 0.23	10.89 ± 0.47	11.08 ± 0.17	10.95 ± 0.35	0.293	0.024*
U6MR-BLW (mm)	17.02 ± 0.71	14.82 ± 0.83	15.36 ± 1.02	14.91 ± 0.49	0.136	0.305
L7-AC (mm)	62.21 ± 0.56	62.41 ± 0.6	62.48 ± 0.5	102.06 ± 39.69	-0.062	0.639
L7-MR (mm)	77.21 ± 1.11	77.93 ± 1.04	76.57 ± 1.25	76.71 ± 1.47	-0.081	0.544
L7-BLW (mm)	8.97 ± 0.87	8.21 ± 0.78	9.28 ± 0.74	8.54 ± 0.94	0.118	0.372
L7MR-BLW (mm)	16.47 ± 0.72	16.95 ± 0.83	17.97 ± 0.69	16.91 ± 0.53	-0.047	0.724
L6-AC (mm)	55.24 ± 0.38	55.97 ± 0.55	56.21 ± 0.67	55.54 ± 0.67	-0.369	0.039*
L6-MR (mm)	59.03 ± 0.66	59.85 ± 0.85	59.29 ± 1.03	60.23 ± 1.22	-0.201	0.127
L6-BLW (mm)	8.29 ± 0.33	8.19 ± 0.38	8.49 ± 0.71	8.55 ± 0.72	0.065	0.627
L6MR-BLW (mm)	12.97 ± 0.42	10.94 ± 0.61	11.02 ± 0.58	10.32 ± 0.82	-0.066	0.619
U7-IM (mm)	60.55 ± 0.8	61.34 ± 0.91	61.29 ± 0.73	61.2 ± 0.65	-0.046	0.727
U6-IM (mm)	55.72 ± 0.16	55.93 ± 0.76	56.54 ± 0.76	55.8 ± 0.65	0.098	0.460
L7-IM (mm)	57.48 ± 0.68	58.08 ± 0.72	58 ± 0.73	58.04 ± 0.59	-0.164	0.214
L6-IM (mm)	50.25 ± 0.55	50.83 ± 0.82	51.05 ± 0.78	49.41 ± 0.87	-0.323	0.029*
∠27/Sagittal (°)	10.44 ± 2.46	14.8 ± 2.49	12.17 ± 2.16	10.84 ± 1.74	-0.123	0.354
∠26/Sagittal (°)	1.41 ± 1.91	5.91 ± 1.83	5.17 ± 1.96	4.79 ± 1.43	-0.222	0.091
∠17/Sagittal (°)	9.35 ± 1.84	13.18 ± 2.56	10.63 ± 3.15	9.79 ± 1.47	-0.023	0.863
∠16/Sagittal (°)	1.77 ± 2.1	4.8 ± 1.95	4.44 ± 1.79	2.7 ± 1.21	-0.153	0.248
∠37/Sagittal (°)	-22.48 ± 2.18	-22.81 ± 2.95	-18.17 ± 4.91	-19.37 ± 2.05	0.182	0.167
∠36/Sagittal (°)	-18.34 ± 1.92	-18.22 ± 1.92	-15.69 ± 1.68	-14.24 ± 0.97	-0. 380	0.037*
∠47/Sagittal (°)	-21.81 ± 1.29	-25.91 ± 2.98	-20.83 ± 2.91	-19.46 ± 2.53	-0.230	0.080
∠46/Sagittal (°)	-17.55 ± 1.65	-18.34 ± 1.56	-16.69 ± 2.13	-14.99 ± 1.89	-0. 471	0. 015*
Coronal: ∠16/OP (°)	1.28 ± 4.54	2.29 ± 4.95	3.05 ± 4.43	0.64 ± 3.66	-0.191	0.143
Coronal: ∠26/OP (°)	-0.6 ± 3.82	3.53 ± 5.75	2.74 ± 4.80	1.61 ± 4.81	-0.245	0.059
Coronal: ∠36/OP (°)	12.61 ± 4.16	13.97 ± 5.15	11.19 ± 4.01	12.94 ± 3.64	-0.046	0.725
Coronal: ∠46/OP (°)	14.25 ± 3.90	13.58 ± 4.80	13.36 ± 3.82	14.84 ± 5.14	-0.089	0.497

Man-Max: the difference between the AG–AG and J-J width; L: left; R: right

^*^p < 0.05

## Discussion

Condylar morphology can be changed as a result of adaptive remodeling to functional or pathological stimuli, even after growth has ceased [22]. Knowledge of the condyle is essential for correctly assessing TMJ status and treatment planning. The TMJ morphology and position of the condylar head vary in diverse sagittal and vertical skeletal patterns [23]. Our research specifically evaluated the difference between the morphological bony changes of the condyle of Class II hyperdivergent females and their association with dentoskeletal characteristics.

Most studies have evaluated the condylar size in some specific plane [2, 24]. By conducting 3D measurements, we found that the condylar volume of the changed group was significantly smaller than that of the normal group. Since function affects form [22], larger condyles seemed to be more resistant to stimulations, while smaller condyles may provide unreliable support for stimulations and thus are prone to pathological changes [25]. Condyle flattening may be the first adaptive alteration to degenerative changes in the TMJ [26]. The resorption of the condylar head probably results in occlusal changes [27]. Osteophyte formation was considered to widen the condylar surface to stabilize and improve loading capacity [28]. Surprisingly, our study showed no significant differences among the condylar volumes of the three changed groups. We speculate that the condyle may diminish after adaptive alteration regardless of the changing form.

**Fig. 9:**
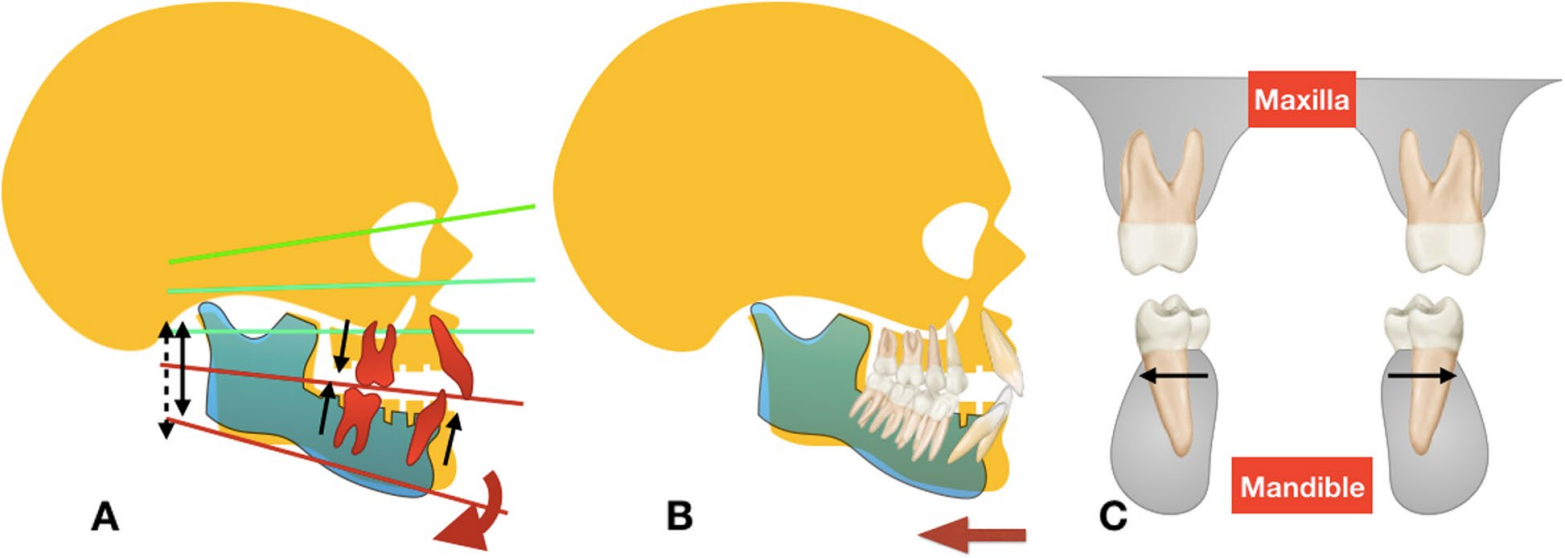
Schematic diagrams of dentoalveolar and skeletal characteristics as condylar volume decreased. A decrease in condylar volume was associated with a retruded and clockwise-rotated mandible with shorter rami. Condylar volume was negatively correlated with overjet, alveolar height of the lower anterior and posterior teeth, sagittal inclinations of the lower teeth, buccal inclination and intermolar width of the mandibular first molars, and width between the corresponding alveolar crests

Our results indicated that condylar volumetric reduction was accompanied by shorter total mandibular length, ramus height, posterior facial height, reduced posterior-to-anterior facial height ratio and steeper occlusal plane, but was not associated with anterior facial height, lower face height, or mandibular body length (Table 4, Fig. 9A). Overall, the mandible displayed clockwise rotation with condylar volumetric reduction, but the maxilla remained relatively unchanged. Ahn et al. found a similar trend in that posterior facial and ramus heights were significantly different between patients with and without degenerative diseases [29]. Nonetheless, Gidarakou et al. found a reduction in both the SNA and SNB angles in patients with TMJ degeneration [30]. The increased alveolar height of the posterior and mandibular anterior teeth may result from compensational extrusion of the teeth after clockwise rotation of the mandible.

According to our results, while the mandible displayed clockwise rotation with condylar volumetric reduction, the occlusal plane and the sagittal inclination of maxillary molars were relatively stable. In contrast, condylar volume and the sagittal angulation of mandibular molars, whether based on the coronal plane or based on the occlusal plane, were negatively correlated. We speculate that when the mandible rotated clockwise, the occlusal force component that made the lower posterior teeth tip mesially would be greater. However, the upper posterior teeth were less impacted because they were relatively more upright in the alveolar bone (Table 5). The mesially tipped lower posterior teeth compensated for the possible deterioration of the sagittal occlusal discrepancy.

Meanwhile, the lower incisors compensatorily protruded to have occlusal contact with the upper incisors, manifesting as sagittal inclination of incisors negatively correlated to condylar volume. However, the compensation was insufficient for the increased overjet caused by the retrusive mandible. In addition, muscular activity should be considered. Changes of muscular pressure were suggested to be associated with alteration of dentition position [31]. Tongue pressure on the lower incisors increased when the mandible retruded, whereas the labial-lingual muscle strength on the upper incisors was almost balanced. Thus in our study, condylar volume was positively correlated with the U1-L1 angle and negatively correlated with the IMPA angle and overjet, but not correlated with the U1-SN angle (Fig. 9B).

No previous study has established a relationship between condylar bony changes and dentoskeletal features in the transverse dimension. Our results implied that the condylar volume was negatively correlated with the arch width of the lower first molars. Moreover, we have evaluated the buccolingual inclination (BLI) of molars separately based on the coronal plane and the occlusal plane. The results showed that the condylar volume was negatively correlated with the BLI on the coronal plane. However, after orientating the functional occlusal plane parallel to the floor, the BLI on the occusal plane were not correlated with condylar volume (Table 6). We assumed that the difference of bilateral occlusal plane would affect the measurement of buccolingual “orthodontic inclination”. The inclination on the coronal plane have neglected the impact of occlusal plane. We hypothesized that when the mandible rotated clockwise, the retropositioned lower arch would be occluded with a wider posterior upper arch. Thus, the occlusal force on the lower molars is more buccally inclined. Meanwhile, the force of tongue muscle pressure on the lower molars relatively increases. Consequently, the buccal inclination on the coronal plane and arch width of the lower first molars increases (Fig. 9C). However, since the clockwise rotation on the y-axis hypothetically impact the vertical position of bilateral molars unequally. The influence was too complicated to consistent, thus when oriented the CT by occlusal plane, such correlation became inconsistent. The basal bone inclination is frequently aligned with the inclination of the mandibular molars [32] and was not affected by occlusal plane. Thus, the width between the buccal alveolar crests in the lower first molar section increases.

Our study established a comprehensive correlation between condylar volume and dentofacial deformities, which could be useful for indicating potential pathological changes in the TMJ. However, the cause-and-effect relationship remains unclear. A homogeneous population may have racial, sex, and skeletal differences affecting the results. This needs to be clarified in future studies. In addition, it was not possible to perform CT scans on more subjects to enlarge the sample size due to ethical reasons; therefore, the results of this study would be better interpreted and supported by new studies.

## Conclusions

Condylar bony changes showed a reduction in volume compared to normal condyles, regardless of the changing forms. Vertically, a decrease in condylar volume was associated with a decrease in rami, posterior facial height, posterior-to-anterior face height ratio, total mandibular length, and an increase in the occlusal and mandibular plane angles, indicating clockwise rotation of the mandible. Meanwhile, the alveolar height of the posterior and mandibular anterior teeth increased, along with a decrease in condylar volume. Sagittally, a decrease in condylar volume was accompanied by a more retruded mandible, mesially tipping mandibular posterior teeth, labially inclined mandibular anterior teeth, and a larger overjet. Transversely, a decrease in condylar volume correlated witharch width, and alveolar crest width of the mandibular first molars.

## Data Availability

The datasets used and/or analyzed during the current study are available from the corresponding author on reasonable request.

## Abbreviations

TMJ: Temporomandibular joint
TMD: Temporomandibular disorder
CT: Computed tomography
CO: Condylion
ICC: Intraclass correlation coefficient
